# Comparative Study Between Two Treatment Regimens of Cisplatin-5-Fluorouracil and Gemcitabine-Cisplatin in Gallbladder Cancer Patients

**Published:** 2017

**Authors:** Daud Ali, Nawazish Alam, Farhat Yasmin, Sajid Ali, Tarique Anwer, Sarfaraz Alam, Sarfaraz Ahmad

**Affiliations:** a *Departmentof Pharmacy Practice, National Institute of Pharmaceutical Education & Research, Hajipur, India.*; b *Collegeof Pharmacy, Jazan University, Jazan, KSA.*; c *Bottomup Technologies, Mahagma, Godda, Jharkhand, India.*

**Keywords:** Gallbladder cancer, Gemcitabine, 5-Flurouracil, Cisplatin, Overall response rate (ORR)

## Abstract

The research study was designed to compare the safety and efficacy of the regimen of 5-flurouracil with cisplatin of investigational armwith the reference regimen of gemcitabine with cisplatin for the treatment of gallbladder cancer.Atotal of 60 patients were enrolled in the study.Out of 30 patients enrolledinarm-A (Gemcitabine withCisplatin) and 30 patients enrolledin arm-B (5-Flurouracilwith Cisplatin) for safety assessment. For the efficacy evaluation total of 16 patients enrolled which is equally divided in both arm.Total 150 cycles of chemotherapy were given to each arm of patients. Both armswere well balanced with respect to age, stage of disease and measurability.The overall response rate (ORR) was 62.5% in arm-A compared to 50% in arm-B (p = 0.34). Whereas 95% confidence interval (CI) for the efficacy was found 46.25-8.74%*vs*32.67-67.32% between arm-A and arm-B.The most prevalent toxicities were found anemia (p < 0.05), neutropenia (p < 0.05) leucopenia (p<0.05) and thrombocytopenia (p < 0.05) and it occurred at a higher rate in arm-B than in arm-A of various grades.There was no statistically significant efficacy & toxicity for gemcitabine and cisplatin with 5-flurouracil and cisplatin however there was an overall more benefit in arm-A patients than arm-B patients.

## Introduction

Cancer is a class of diseases characterized by uncontrolled cell division and the ability of these cells to invade other tissues, either by direct growth into adjacent tissue through invasion, or by migration of cells to distant sites by metastasis (1, 2). This unregulated growth is caused by series of acquired or inherited mutations to deoxyribonucleic acid within the cells, damaging genetic information that defines the cell functions and removing normal control of cell division (3).

Cancer of the gallbladder is a disease in which cancer cells are found in the tissues of the gallbladder (4). Majority of the gallbladder tumors are found in glandular tissue within the gallbladder (Adenocarcinoma). Others originate in the connective tissue of sarcoma or other tissues of squamous carcinoma (5). 

Adenocarcinoma of the gallbladder is uncommon in the western world, with approximately 5,000 cases of gallbladder carcinoma annually in the USA(2003) worldwide (6,7).The highest prevalence of gallbladder cancer is seen in Israel, Mexico, Chile, Japan, and among Native American women, particularly those living in New Mexico (8,9,10).

Incidence of gall bladder cancer of Gangeticriver regions of Vaishali, rural Patna and Varanasi to be around 20-25/100,000populations in India. Gallbladder cancer, the fourth common cancer in Delhi is the highest among the Asian registries and third highest among the registries reported from world (11). Compared to this, the rate in Bangalore was just 0.5/100,000 population and 12.5/100,000 in Delhi (12).As per Indian registriesthe incidence of gallbladder cancer was found second highest (6%) in females of metropolitan cities and age standardized rate was found 7.4 (13).

The river water in the region is polluted due to flow of affluent from the neighboring industries, especially the tanneries. Cadmium is banned the world over. The ratio of incidences of the cancer in North and South India was at an extreme 15:1(14).

## Material and Methods

The clinical study was carried out in accordance with the Basic Principles defined in the International Conference on Harmonization ‘Guidance for Good Clinical Practice’ and the principles enunciated in the World Medical Association’s Declaration of Helsinki. The study was carried out inoutpatient department and in patient department at Mahavir cancer Institute and research Centre Phulwari Sharif Patna, Bihar, India.

This study was a prospective, open label, randomized, comparative study to evaluate the safety, efficacy and toxicity profile between two treatment regimens (gemcitabine with cisplatin in arm –A and 5-flurouracil with cisplatin in arm – B) used in treatment of gall bladder cancer of inoperable stage IV.The Institutional Ethical Committee (IEC) of mahavir cancer institute,Phulwarisharif, approved the study as well as the research protocol.

Data relevant to study were collected from, the patient’s profile of medical record room and nursing station of inpatient department, those will be planned for regimen A or regimen B chemotherapy of carcinoma of the gallbladder. Total of 60 patients taken, 30 for each regimen for toxicity and safety study, and out of which 16 patient wereequally divided in two groupsfor efficacy study.

*Inclusion criteria*

- Histological or cytological confirmed or metastatic adenocarcinoma of the gall bladder carcinoma
- Age between 18 to 70 years.
- At least 1 measurable lesion according to the response evaluation criteria in solid tumours (RECIST)
- Patient at least completed 2 cycles of chemotherapy.
- Both sexes (male and female)
- Adequate bone marrow function (absolute neutrophil count) [ANC] ≥ 1500Cu mm Platelet count ≥ 10,0000Cu mm
- Hepaticfunction(aspartameaminotransferase,alanine N-aminotransferase) [AST/ALT] ≤times the upper limit of normal
- The upper limit of normal [ULN] Bilirubin≤2.0 times the ULN,
- Renal function (serum creatinine≤1.5 times the ULN),
- TNM clinical classification stage more than IVA/IVB

*Exclusion criteria*

- Organ failure
- HIV/Hbs Ag +ve
- Severe bone marrow suppression
- Pregnant and lactating women
- Patients who go prior to chemotherapy
- Allergic or anaphylactic like reaction

*Patient enrolment*

A total of 60 patients of gallbladder cancer, equally (30+30) from both arm of either sex who fulfilled the study criteria, were enrolled into the study after the nature of the study was explained to them and their written informed consent was obtained.

Before the starting of chemotherapy,baseline status of complete blood count, renal function test and liver function test were performed, and results of all hematological tests were recorded. Likewise all the hematological test were also performed before starting next cycle of chemotherapy,this results allow whether the next cycle of chemotherapy should be allow or not and result of all these test were also recorded.

**Table 1.** Hematological toxicity according to NCI-CTC

**Type of toxicity**	**Group A**		**Group B**		
	**n**	**%**	**n**	**%**	
**Anemia**					
Grade I	52	34%	69	46%	0.021*
Grade II	11	7.3%	26	17.3%	0.032*
Grade III	13	8.6%	11	7.3%	0.064
Grade IV	4	2.6%	7	4.6%	0.027*
**Leucopoenia**					
Grade I	3	2%	11	7.3%	0.032*
Grade II	7	4.6%	8	5.3%	0.046*
Grade III	4	2.6%	4	2.6%	0.018*
Grade IV	1	0.6%	4	2.6%	0.019*
**Thrombocytopenia**					
Grade I	13	8.6%	9	6%	0.062
Grade II	10	6.6%	8	5.3%	0.069
Grade III	4	2.6%	2	1.3%	0.065
Grade IV	0		3	2%	0.047*
**Neutropenia**					
Grade I	2	1.3%	7	4.6%	0.031*
Grade II	17	11.3%	14	9.3%	0.071
Grade III	5	3.3%	8	5.3%	0.034*
Grade IV	1	0.6%	8	5.3%	0.025*

n= number of chemotherapy cycle,

* p<0.05

**Table 2:** Response according to RECIST Criteria

**Efficacy parameter**	**Arm-A** **n, %**	**Arm-B** **n, %**	**P value**
Response	5, 62.5%	4, 50%	0.34
Complete response	1, 12.5%	0	0.5
Partial response	4, 50%	4, 50%	0.38
Stable disease	0	3, 37.5%	0.1
Progression of disease	3, 37.5%	1, 12.5%	0.25
95% Confidence Interval	46.25-8.74	32.67-67.32	NA

n= number of subject

*Collection of Data*

All the required data relevant to the study, patients including demographic details such as name, sex, age, occupation, educational status; clinical data such as diagnosis, clinical condition; therapeutic data such as name of the drug, dose, route, frequency, duration of therapy and other relevant details were collected from treatment chart, patient’s case note, and laboratory investigational reports.

*Pretreatment evaluation and treatment plan*

Pre-treatment evaluation included complete medical history, physical examination, and evaluation of performance status, chest radiograph, and diagnostic studies for disease assessment such as ultrasound of the abdomen or CT scan.

The treatment plan involved administration of gemcitabine 1g/m^2^over 30-min of intravenous infusion on days 1 and 8. Administration of cisplatin 75mg/m^2^ was given intravenously over 30 min on day 1. Treatment cycles were repeated every 21 days provided the patient had recovered from any drug-related toxicity associated with the previous course. The treatment course, toxicity grades were reviewed and dosage for the next cycle was modified according to the followed schedule: dose of gemcitabine was reduced by 20% for grade IV neutropenia associated with fever or infection or lasting more than 7 days, absolute neutrophil count of less than 1,000/mm^3^ lasting beyond day 21 of the treatment course, platelet nadir of less than 25,000/mm^3^ or any grade III-IV visual toxicity other than nausea and vomiting. Cisplatin dose was adjusted according to the serum creatinine value. If a patient had any toxicity that required a delay in the next treatment course, dosage in the subsequent cycle was decreased by 20%.Patients were continued on therapy until complete response or disease progression was documented or until unacceptable toxicity occurred. Patients were withdrawn from treatment if there was greater than 2 weeks delay in treatment because of toxicity. 

*Assessment of objective response*

Objective tumor assessments were performed according to response evaluation criteria in solid tumor (RECIST).The primary study end point was objective response rate. Complete response (CR) was defined as the disappearance of all clinical evidence of tumor for a minimum of 4 weeks during which time the patient was free of all symptoms related to cancer. Partial response (PR) was defined as a> 50% decrease in the sum of the products of the longest perpendicular diameters of all measurable disease with no new lesions appearing and none progressing for at least 4 consecutive weeks. Patients were rated progressive (PD) if any new lesion appeared, or tumor size increased by 20% over pre-treatment measurements, or in case of a deterioration in clinical status that was consistent with disease progression. Patients who failed to meet the criteria of CR, PR or PD and who remained on study for at least two months were classified as having stable disease(SD).In case of PR or CR, a second assessment four weeks later was required for confirmation of response.

*Statistical Analysis*

The data collected to compare the Safety, Efficacy and toxicity between Arm-A and Arm-B. Values were expressed as Mean ± Standard deviation or as percentages. Comparison of the mean values within the group was done using Z-test for hematological toxicity, Fisher exact test for efficacy, and chi-square test for non-hematological toxicity respectively. Statistical analysis was performed by SAS software version 9.1.

## Results

Total 60 patients were enrolled in this study, between the period of December 2008 to May 2009 all the patients divided in two Arms, Arm-A and Arm-B in equal number, treatment given to all the patients according to randomization schedule. All enrolled patients completed the study, and taken at least 5 cycle of chemotherapy.

*Patient’s characteristics*

Arm-A:In the age group distribution, the mean age was observed 50(±14) years having range of 24 to 72 years. The number of patients segregated in various age groups of 70-80 years, 60-70 years, 50-60 years, 40-50 years and 20-30 years was 1, 3, 12, 10 and 1 respectively.

Arm-B:In the age group distribution, the mean age was observed 55(±12) years ranging from 35 years to 65 years. The number of patients segregated in various age groups of 60-70 years, 50-60 years, 40-50 years and 30-40 years was 9, 12, 7, and 2 respectively.

Gender distribution in Arm-A:Out of 30 patients,26 female subjects (86.6%) and 4 male subjects (13.4%) were enrolled in arm A.

Gender distribution in Arm-B:Out of 30 patients,27 female subjects (90%) and 3 male subjects (10%) were enrolled in arm B.

*Toxicity*

The bone marrow suppression (anemia, neutropenia,thrombocytopenia,and leucopoenia) was more pronounced in arm-B compared with arm-A.According to National cancer institute common toxicity criteria of (NCI-CTC)was gradeI,II,IIIand IV anemia was observed 46%,26%, 11% and 7% respectively of cycles of chemotherapy. In Arm-A grade I, II, III and IV anemia toxicity was observed 34%, 73%, 86% and26% of patients respectively. The entire grade –III & IV types of anemic patients were received blood transfusion.GradeI&IV thrombocytopenia was more frequently in the Arm-B chemotherapy cycle (7.3%and4% respectively) compared with the with the Arm-A (2% and 0.6% respectively).Grade IV thrombocytopenia in Arm-B chemotherapy cycles were observed in 2% cycle of all the cycles, while in Arm-A patients grade I, II and III observed (8.6%, 6.6% and2.6% respectively)

Grade I, III and IV types also occurred more frequently in Arm-B patients (4.6%, 5.3% respectively) than in the Arm-A (1.3%, 3.3% and 0.6% respectively).

Grade I, II, III and IV toxicity were listed in Table 1. no significant differences between the two treatments arms reported of serious non-hematological toxicities. The incidences of grade IV Hepatotoxicity 2% were more in group-B cycles of chemotherapy in comparison to group-A cycles of chemotherapy.

Grade II, III & IV Nephrotoxicity (1.3%, 2% and 2.6% respectively) were observed in group-A in comparison to group-B(none of them appear toxicity).Grade II, III & IV types of peripheral neuropathy (2%, 1.3% and 1.3% respectively) were observed in group-A cycles of chemotherapy in comparison of group-B. Grade II, III & IV types of constipation (2.6%, 2.6%, and 2.6% respectively) were observed while in group-B none of them were had toxicity.

In group-B for mucositis were with grade II, III & IV (1.3%,1.3% and 2% respectively)were observe while in group-A cycles of chemotherapy were found in grade II, III,( 2%, 3.3% respectively). Grade II, III, IV (3.3%, 4.6%, and 4% respectively) nausea and vomiting were found in group-A cycles of chemotherapy while in group-B cycles of chemotherapy were found to be grade III, IV (4.6%, 0.6% respectively).

*Efficacy*

In group A, all 8 patients had measurable disease. According to the investigator’s assessment 1 patient had a complete response, 4 patients had a partial response, none of them had stable disease and 3 had disease of progression, a response rate in evaluable patients of 62.5%[95% of confidence interval(CI) was found to be 46.25-78.74].

In group B, all 8 patients had measurable of disease. According to the investigator’s assessment, four patients had partial response, three had stable disease and one had disease of progression, giving a response rate of 50% [95% of confidence interval (CI) was found to be 32.67-67.32]. Efficacy data are given in the Table 2.

## Discussion

In this study gemcitabine with cisplatin was taken as the control arm because prior trial had shown a superior over cisplatin with 5-flurouracil according to response rate (15, 16). The results of patients treated with arm-B in this study (response rate of approximately 50%) are comparable with other studies conducted with arm-A as the control arm. In the study no statistically significant difference (P = 0.34) was seen in response rates. The response rate, was slightly higher for arm-A than arm-B (62.5%, *Vs* 50% respectively).

Toxicity was observed more frequently in Arm-B as compared to Arm-A. Previous study also supported that gemcitabine with cisplatin is more toxic than cisplatin with 5-fluorouracil(17, 18). The incidence of anemia grade III-IV (11%) in arm-A while in arm-B it was observed (18%) in comparison with the arm-A. The incidence of thrombocytopenia was observed in arm-B with grade-III-IV (3.3%) while in arm-A patients were observed same toxicity with (2.6%). Thrombocytopenia resulted in a large percentage of patients experiencing dose reduction or omission of gemcitabine and occurred most commonly on day 15. Nearly one half of the gemcitabine dose reduction and two thirds of the gemcitabine dose omission on day 15 were a result of thrombocytopenia. Such reduction/omission dictated by thrombocytopenia has not been seen with single agent gemcitabine. Leucopenia with grade III-IV toxicity (5.2 %) was found more common in arm-B in comparison on to arm-A (3.2%). Non-hematological toxicity was more common in arm-A in comparison to arm-B. Grade III-IV Hepatotoxicity was observed in arm-B in comparison to arm-A (1.3%). Grade III-IV Mucositis toxicity (3.3%) was similar in both arms. Peripheral neuropathy was more common in arm-A patients while none of the patients in the arm-B had the same toxicity. Constipation was also common among patients in arm-A in comparison to none of the patients in arm-B reported constipation in any cycles of chemotherapy. Hyperglycemia of grade III-IV (3.3%) was observed in arm-A where none of the patients had same in arm-B during all the cycles of chemotherapy. All the hyperglycemic patients with grade III-IV toxicity were prescribed with anti-diabetic drugs. 

The limitation of the study was unable to find clinical significance for efficacy. Sample size was not adequate enough to detect clinical significance in response rate between two arms due to time constrain and insufficient budget. To get the power of 90% for having a clinical significance of response rate we need to have nearly 512 subjects in two arms.

## Conclusion

The present research work was “A prospective cohort study to assess the efficacy, safety, and toxicity of gemcitabine-cisplatin in comparison to 5-fluorouracil-cisplatin in treatment of gallbladder cancer”. The overall response rate of gemcitabine-cisplatin (62.5%) as compared tocisplatin-5-fluorouracil (50%) was statistically not significant (p=0.34). The efficacy of gemcitabine-cisplatin in the present study was higher as compared to cisplatin-5-fluorouracil. However, previous studies have shown significantly higher efficacy of gemcitabine-cisplatin as compared to cisplatin-5-fluorouracil. Despite the lack of statistical significance not observed in the present study may be due to inadequate sample size and time bound research work of postgraduate thesis.As far as safety concerned, 5-fluorouracil with cisplatin seems to be safer than gemcitabine with cisplatin. We did not observe any significant difference of toxicity properties between two regimens. We suggest that this study can be conducted on large sample size in future for getting significant result.
